# Disordered Eating Behaviors in Children and Adolescents with Type 1 Diabetes: Age-Specific Challenges and Management Insights

**DOI:** 10.3390/children13040474

**Published:** 2026-03-29

**Authors:** Aristeidis Giannakopoulos, Ioanna Kosteria, Alexandra Efthymiadou, Eirini Kostopoulou, Natasa Chrysanthakopoulou, Dionisios Chrysis

**Affiliations:** 1 Division of Pediatric Endocrinology, Department of Pediatrics, University Hospital of Patras, Rio, 26504 Patras, Greeceeirini.kost@gmail.com (E.K.); dchrysis@upatras.gr (D.C.); 2 Department of Endocrinology, Growth & Development, “P&A Kyriakou” Children’s Hospital, 11527 Athens, Greece

**Keywords:** type 1 diabetes, disordered eating behavior, eating disorders, pediatric diabetes, nutrition

## Abstract

Type 1 diabetes is the most common autoimmune endocrine disorder in children and adolescents, with incidence rising worldwide. Its management demands comprehensive care encompassing glucose monitoring, insulin therapy, and individualized nutritional education to support glycemic control and overall health. Establishing normal eating patterns is pivotal not only for prandial euglycemia but also for reducing the risk of disordered eating behaviors and eating disorders that are more frequently observed in youth with diabetes. Because eating patterns and self-management capabilities vary by developmental stage, interventions must be tailored to the physical, psychological, and social context of each age group. Screening tools such as the Diabetes Eating Problem Survey—Revised (DEPS-R) enable timely identification of at-risk individuals. In this scoping review we present the data from the literature regarding the eating patterns and deviations from infancy to adolescence, report the complications and discuss the challenges and insights for their management.

## 1. Introduction

Type 1 diabetes (T1D) is the most prevalent autoimmune endocrine disorder affecting children and adolescents globally, with an estimated 1.2 million individuals under 20 years of age diagnosed worldwide [1]. The most significant increase is observed in the youngest subgroup (0–4 years), where incidence rates escalated from 5.8 to 19.9 per 100,000 person-years over a 20-year period [2].

Effective management of T1D requires a comprehensive approach that includes regular glucose monitoring, accurate insulin administration, evaluation of carbohydrate and caloric intake, and tailoring the treatment plan to fit family routines, dietary preferences, and daily activities. T1D treatment in children is also affected by various factors, such as stages of growth and development, psychological attributes, coexisting health conditions, family dynamics, and the quality of care provided outside the home [3]. With advancing age, self-management of T1D starts having a central role, encompassing symptom control, treatment adherence, addressing physical and psychosocial effects, and making necessary lifestyle changes [4]. Therefore, a structured diabetes education approach that integrates all the aforementioned factors is essential.

Nutritional education is a cornerstone in the comprehensive management of T1D, as it empowers young patients and their families to make informed dietary choices that support glycemic control and overall health. Prandial glucose control contributes substantially to HbA1c levels in T1D, though its relative contribution varies depending on overall glycemic control. Data from continuous glucose monitoring in adults with T1D show that postprandial hyperglycemia accounts for about 24% of the variance in HbA1c, while fasting hyperglycemia contributes around 32%, with both factors explaining a significant portion (74%) of HbA1c variability. The relative impact of prandial glucose tends to be greater when HbA1c is closer to target levels implying that controlling prandial glucose excursions remains crucial, especially for improving HbA1c near target ranges [5]. The International Society for Pediatric and Adolescent Diabetes (ISPAD) and the American Diabetes Association (ADA) emphasize individualized nutrition therapy tailored to age, developmental stage, and family context, highlighting its importance in both preventing acute metabolic complications and minimizing long-term risks such as cardiovascular disease [6,7]. Evidence from high-quality studies demonstrates that structured nutritional interventions improve adherence to glucose monitoring and insulin dosing, reduce glycemic variability, and enhance quality of life [8]. The emphasis on nutrition in the management of T1D can also pose challenges for children, adolescents, and their families as they strive to regulate prandial glucose levels. This increased focus may elevate the risk of developing disordered eating behaviors (DEBs). Such behaviors, compounded by additional factors, have the potential to progress into more severe eating disorders (EDs), particularly given that school-age and adolescence constitute developmental periods of heightened vulnerability due to ongoing mental and emotional maturation [9].

Consequently, nutritional education plays an important role in preventing DEBs and should involve continuous support and integration of behavioral strategies to engage the child and family, fostering sustainable healthy eating habits [10,11]. The aim of this scoping review was to synthesize current evidence on disordered eating behaviors in pediatric T1D from infancy to adolescence, identify age-specific risk factors and complications, and propose practical management strategies.

## 2. Methodology

**Figure 1:**
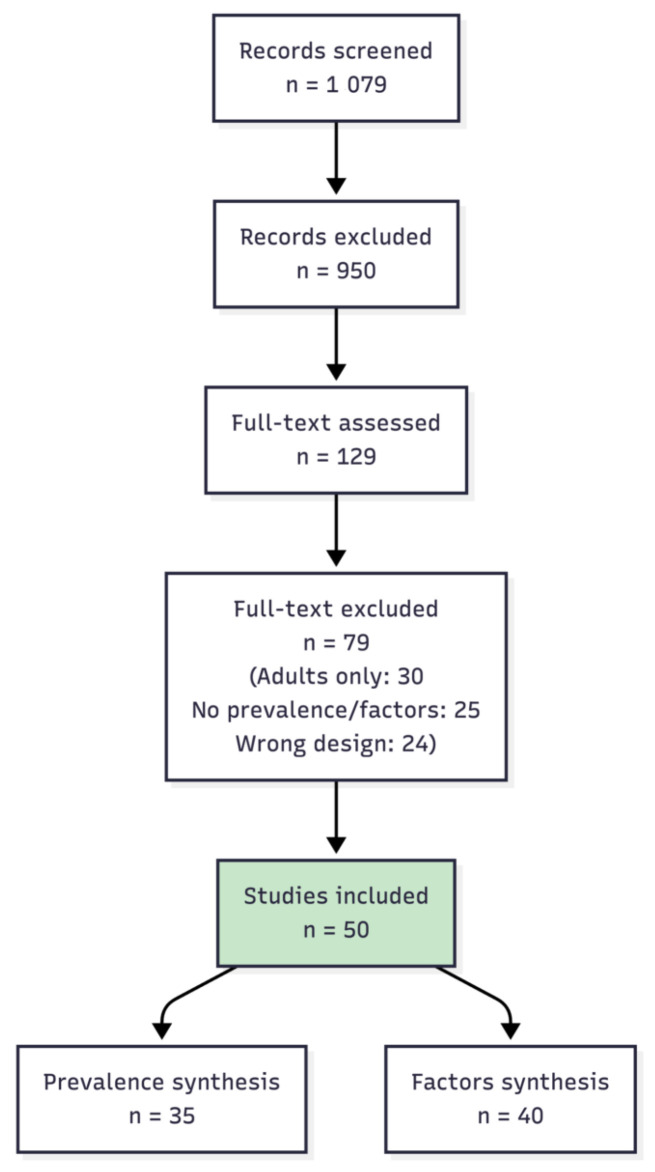
PRISMA-ScR flow diagram illustrating the study selection process. Records were identified through database searching, screened by title and abstract, assessed by full-text review, and included based on eligibility criteria requiring reporting of DEB/ED prevalence and associated factors.

This scoping review adhered to the Preferred Reporting Items for Systematic Reviews and Meta-Analyses extension for Scoping Reviews (PRISMA-ScR) guidelines. A protocol was developed prior to data extraction; registration was not completed, consistent with current practice for scoping reviews. Studies were included if they reported prevalence rates or predisposing/risk factors for EDs or DEBs in children (0–12 years) and adolescents (13–18 years) with T1D. Eligible designs included observational studies (cohort, cross-sectional, case-control), reviews, and qualitative research. Excluded studies: adults only (>18 years), type 2 diabetes, non-English publications, and non-peer-reviewed sources. Searches covered PubMed/MEDLINE, Embase, Web of Science, and Scopus from inception up to and including 31 January 2026. Keywords combined MeSH terms: (“eating disorder*” OR “disordered eating” OR DEPS-R OR “insulin omission”) AND (“type 1 diabetes” OR T1D) AND (“child*” OR “adolescen*” OR pediatric*). Duplicates removed via EndNote (Figure 1). Data extracted into a piloted form capturing author/year, study design, population (age, sample size, T1D duration), prevalence estimates, predisposing factors (e.g., female sex, glycemic control, family environment), and tools (e.g., DEPS-R, SCOFF). No formal quality appraisal, per scoping review standards, though limitations noted. Findings synthesized narratively by prevalence and predisposing factors. A potential selection bias may have been introduced due to the predominance of literature from European and North American contexts, reflecting the review’s restriction to studies published in the English language.

## 3. Eating Behavior Deviations in Pediatric T1D

There is broad consensus that individuals with T1D are at heightened risk of developing DEBs, characterized by maladaptive eating attitudes and practices such as unhealthy weight-control behaviors, binge eating, chronic dieting, self-induced vomiting, overeating, and the violation of dietary restrictions [12,13,14]. Specific aspects of daily T1D management, including the continuous focus on food and eating, the imposition of dietary restraints, the necessity of eating to correct hypoglycemia, and insulin-associated weight gain, are believed to exert an iatrogenic influence on the emergence of these behaviors [15].

Emotional eating displays a pattern that consists of eating in response to negative emotions such as anxiety, sadness, loneliness, anger, and depression. In the context of pediatric T1D, emotional eating represents a maladaptive coping strategy where individuals consume food in response to psychological distress, including emotions related to the burden of diabetes management [16]. Within the general population, emotional eating has been identified as a risk factor for the onset of binge eating behaviors [17]. Emotional eating is significantly more common among adolescents with T1D, especially older teens, than it is in those without diabetes. This behavior is also positively linked to higher HbA1c levels [18]. Furthermore, emotional eating is strongly linked to other DEBs, with affected individuals showing higher emotional eating scores than those without such tendencies [16].

DEBs encompass a spectrum of atypical eating-related actions, including food restriction, selective nutrient manipulation, meal skipping, excessive exercise for weight control, binge eating, self-induced vomiting, and the use of diuretics or laxatives [12,19]. These behaviors tend to occur with lower severity and may not meet full diagnostic criteria required (for example the DSM-5 criteria for anorexia nervosa needs restriction of energy intake relative to requirements, intense fear of gaining weight and disturbance in body image) for a formal diagnosis of an ED but nonetheless impact health and functioning [20]. DEB prevalence is notably higher among youth with T1D compared to their non-diabetic peers, with reported rates reaching up to 38% in females and 16% in males [21,22]. A study involving 770 adolescents with T1D aged 11–19 reported a DEB prevalence of 27.7% in females and 8.7 in males [23]. DEB progressively increases from early childhood to late adolescence, and its prevalence exhibits a marked increase in relation to both advancing age and higher body weight, rising from 7.2% among underweight individuals to 32.7% in those with obesity [15]. Adolescents exhibiting DEB demonstrated elevated HbA1c levels, increased body mass index (BMI), and more pronounced emotional and behavioral difficulties relative to those without eating deviations [23]. Family environment has a significant role in DEB development. Lower priority placed on family meals, less parental support of healthy eating, more food restrictions in the household, and diabetes-specific family conflict, all associate with elevated DEB risk [24]. Some evidence suggests that multiple daily injections versus insulin pump therapy may be associated with DEB, though findings remain inconclusive [25]. Clinical EDs represent psychiatric diagnoses characterized by severe disturbances in eating behavior, attitudes toward food and weight, and body image perception [26,27]. In pediatric T1D populations, recognized EDs include Anorexia Nervosa, Bulimia Nervosa, Binge ED, Avoidant Restrictive Food Intake Disorder and ED Not Otherwise Specified. Anorexia Nervosa is characterized by restricted food intake leading to significantly low body weight, intense fear of weight gain, and disturbed body image [27,28], Bulimia Nervosa features recurrent binge eating episodes followed by compensatory behaviors such as self-induced vomiting, laxative misuse, or excessive exercise. Avoidant/Restrictive Food Intake Disorder (ARFID) is characterized by persistent avoidance or restriction of food intake that is not motivated by body image disturbance or fear of weight gain, but rather stems from sensory-based aversions, lack of interest in eating, or apprehension regarding aversive consequences. Binge ED involves recurrent binge eating without regular compensatory behaviors and ED not otherwise specified encompasses clinically significant eating disturbances that don’t meet full criteria for other specified disorders [12,29]. ED are more common in individuals with T1D compared to their peers without diabetes with a prevalence that ranges from 20.9% to 33.3% in various T1D populations [30]. A meta-analysis of 45 studies by Niemelä et al. reported a pooled prevalence of ED symptoms at 24%. The authors observed that in studies with a higher proportion of female participants (>58%), the prevalence of ED symptoms increased to 30% [31]. A longitudinal study examining the prevalence of EDs in a cohort of 126 girls with T1D, spanning from a mean age of 11.8 ± 1.5 years to 23.7 ± 2.1 years, found that 32.4% met diagnostic criteria for an ED, with an additional 8.5% exhibiting subthreshold ED symptoms. The authors concluded that EDs are prevalent, persistent, predominantly affect females, and may develop later into adulthood [9]. A Swedish nationwide study found that children and adolescents with T1D are over twice as likely to develop psychiatric disorders as their peers and siblings without diabetes, with EDs being the second most common after substance abuse [32].

Prandial insulin omission or restriction represents a significant behavioral deviation related to eating with potentially detrimental effects on the management of T1D, due to the risk of diabetic ketoacidosis [15]. Insulin omission in children and adolescents with T1D has multiple causes, including psychological, behavioral, and physiological factors [33]. An Italian national study has documented a rate of 42.4% of insulin misuse among adolescents with T1D [34]. However, gender disparities are evident, as approximately 10.3% of adolescent females report intentionally skipping insulin and 7.4% report taking less insulin for weight management, whereas such behaviors are reported infrequently among males. These disordered eating patterns showed no significant correlation with demographic factors—including age, parental education, or ethnicity—but were consistently linked to higher levels of weight dissatisfaction, whereas the relationship with BMI remained variable [22]. In general, unhealthy weight control practices were observed in 37.9% of female participants and 15.9% of males and this is observed because weight gain often occurs following T1D diagnosis and insulin initiation—patients typically experience weight loss before diagnosis due to uncontrolled hyperglycemia, then regain (and sometimes exceed) previous weight with insulin treatment [35]. This behavior was also linked to psychological factors beyond emotional eating, including disease denial, self-destructive and suicidal ideation, and fear of severe hypoglycemia [36]. Mental health conditions such as anxiety and depression significantly contribute to insulin omission by increasing vulnerability to maladaptive coping strategies [35], alongside fears of hypoglycemia and interference with daily activities [35,37]

Finally, it is important not to overlook the growing number of people diagnosed in the preclinical stages of T1D. While eating habits or EDs in this group have not yet been specifically studied, increased stress levels especially in parents of children with T1D have been documented [38,39]. Published guidelines on the monitoring of people with positive antibodies underline the importance of psychological support and nutritional education [40]. In this context, it may be equally important to be vigilant for disordered eating habits, such as omission of carbohydrates, to prolong the insulin-free period.

## 4. Age Related Eating Patterns and Disorders

Managing T1D in infants and toddlers is challenging because young children cannot reliably communicate hypoglycemia symptoms, and their frequent feeding patterns contribute to unstable glucose levels. Toddlers seeking autonomy may resist food or become picky eaters, further increasing glycemic variability. These behavioral and dietary challenges require caregivers to set limits and adapt to daily routines. Overall, caring for young children with T1D, places significant emotional and practical demands on families, since caregivers must oversee all aspects of diabetes care and therefore the difficulties and psychological distress from the everyday routine may sometimes cause disruptions in bonding impairing trust and complicate further the management of T1D [41,42,43].

During preschool and early elementary years, T1D management in children becomes highly complex, requiring attention to physiological, developmental, and psychosocial factors. Interventions must address the child’s medical and emotional needs, as well as parental stress and family challenges. The ability of young children to recognize or communicate hypoglycemia symptoms may be still limited [44], and parents find it often difficult to distinguish these from typical behaviors like tantrums or refusal to eat [45]. Balancing discipline with understanding the burden of T1D is a common challenge for parents, with some adopting a lenient approach to alleviate the child’s difficulties [46]. In addition, physical activity levels tend to be lower in children diagnosed with T1D before the age of seven compared to peers without diabetes [47], and this is often due to parental concerns about activity-induced hypoglycemia or decreased capability to provide the adequate insulin adjustments needed for optimal glycemic control during increased physical activity [48].

Transition to school-aged children with T1D often means taking on new self-management responsibilities and concurrently depending on parents for help with complex tasks like carbohydrate counting and insulin dosing. This reliance and lack of confidence can limit their independence and participation in social activities. Many children also experience difficult emotions—anger, sadness, and frustration—due to dietary restrictions and the impact on their social lives, placing them at greater risk for depression, which can further hinder diabetes control [49,50]. This phase demands special attention because, as children strive for independence in managing their diabetes, some may intentionally alter their eating behaviors in search of solutions they perceive as helping with glycemic control, i.e., skipping meals to avoid using insulin, limiting the variety of food choices to avoid calculations, choosing packaged pre-calculated meals, or skipping insulin to avoid hypoglycemia. This is particularly common within the school setting, where there is often a shortage of trained personnel able to successfully support the child with T1D, but also adequate time and space allocation [51]. This gap highlights the importance of targeted education and the role of specialized staff, such as school nurses, to provide appropriate support and respond effectively to emergencies [52].

Adolescence is a period of increased risk-taking, often involving experimentation with drugs or alcohol, and for those with T1D, this translates to possible acute complications and higher hospitalization rates [53,54]. Regarding their dietary patterns, adolescents with T1D often do not meet ADA nutrition recommendations, with low fruit and vegetable intake and high saturated fat consumption [55,56]. Adolescents with T1D are also 2.5 times more likely to develop EDs compared to their peers with the average age of onset occurring around 14 years [35,57]. Several factors contribute to this heightened vulnerability, including the profound physical and hormonal changes associated with puberty, intensified concerns regarding body image and weight, greater susceptibility to peer influence, and exposure to social pressures, such as those arising from social media or participation in sports that emphasize thinness. In addition, psychological risk factors—such as perfectionism, low self-esteem, and mood disturbances—become increasingly salient during adolescence and interact with both environmental and biological changes to further elevate the risk [58] Adolescents exhibit increased autonomy regarding dietary choices and may demonstrate a greater propensity to engage in dieting behaviors, which significantly elevates the risk of developing EDs. Research has shown that dieting or insulin omission for weight control is especially prevalent among teen females and correlates with an increased likelihood of developing an ED [23].

## 5. Complications

In children and adolescents with T1D, eating behavior deviations and EDs result in significant and specific complications that differ from those in the general population due to the interaction with diabetes management. The various eating disturbances described above carry serious medical and psychological consequences. Adolescents with EDs are more prone to diabetic nephropathy and demonstrate poorer glycemic control [59]. They also experience higher rates of diabetic ketoacidosis [60]. Mortality rates are elevated with the standardized mortality ratio for concurrent T1D and anorexia nervosa being 14.5, compared to 4.06 for T1D alone and 8.86 for anorexia nervosa alone [61].

Insulin omission or restriction, in addition to poorer glycemic control, may lead to diabetic ketoacidosis (DKA), which can be life-threatening, and a major factor of increased mortality [27,60]. Beyond the chronic hyperglycemia, the risk for diabetic ketoacidosis (DKA), shows a calculated incidence of 112.5 per 1000 patient-years in those with insulin restriction behavior versus 30.8 in those without [62]. There are also a higher prevalence and earlier onset of diabetes-related microvascular complications of retinopathy, nephropathy, and neuropathy that are significantly correlated with the duration and severity of insulin omission behaviors [27,33,60].

Depending on the age of presentation or worsening, nutritional imbalances from eating deviations, including restrictive diets such as low-carbohydrate regimens, may have a major impact on growth and development in pediatric T1D patients. Growth retardation is linked to insufficient energy intake and altered hormonal milieu influenced by poor insulin treatment adherence. Severe malnutrition may lead to electrolyte imbalances, impaired cognition, postural hypotension, bradycardia, and poor bone health [63]. Some children may shift toward selective nutrient manipulation such as diets high in fats in an effort to avoid blood sugar spikes after eating carbohydrates. However, this can result in persistent hyperlipidemia, characterized mainly by elevated LDL levels, which may require statin therapy if the condition is not reversed by dietary adjustments.

Insulin omission as weight control strategy (diabulimia) has been found to have direct effect on body composition and image, with central fat accumulation, higher waist circumference and lower muscle mass due to protein catabolism [59]. On the other hand, binge eating or excess snacking as a means to treat or prevent hypoglycemia, strengthened by fear of hypoglycemia and ease of insulin administration through pumps, are contributing factors to the emerging increase of overweight and obesity in children with T1D (diaobesity), leading in turn to poor glycemic control and body image dissatisfaction [9,64,65].

EDs in T1D are also frequently accompanied by increased anxiety and depression, creating a vicious cycle that further complicates disease and ED management. Depression and anxiety are risk factors for the development and persistence of DEBs [24,66].

In summary, children and adolescents with T1D who engage in eating deviations or develop EDs face increased risks of acute metabolic crises, chronic microvascular complications, impaired growth, and mental health challenges, all contributing to increased morbidity and mortality.

## 6. Early Identification, Prevention, and Management

Alterations in eating behaviors among children and adolescents with T1D may emerge progressively throughout the course of disease management. Such patterns often remain unnoticed by caregivers, particularly when they appear to have an ostensible beneficial impact on glucose control that may lead caregivers to overlook the potential long-term metabolic consequences of these maladaptive dietary adjustments [11,67].

There are two primary reasons why it is critical to monitor altered eating behaviors closely from the early stages of diagnosis in preschool children, and throughout school years and adolescence. First, the increasing incidence of T1D in younger populations, places early emphasis on prandial glucose control during a developmental stage when dietary habits and eating patterns are being established, making individuals more vulnerable to deviations in eating behavior. Second, evidence from the literature suggests a potential progression from emotional eating to DEBs, and in some cases, to full EDs among children and adolescents with T1D. Subjects with DEB show significantly higher emotional eating scores than those without DEB, indicating that emotional eating may be a risk factor or precursor for DEBs in this population [16,18,66].

Preventing the development of DEB requires several precautions. While nutritional education remains a cornerstone of diabetes management in children and adolescents, it is equally important to recognize that occasional episodes of postprandial hyperglycemia or hypoglycemia are common and often unavoidable in T1D care, especially in younger ages. Various factors—such as increased physical activity, minor insulin dosing errors, and stress—can influence post-meal glucose levels [10]. The primary goal should be to preserve psychological balance, enabling eating to remain a natural physiological process for children with diabetes, while also acknowledging that individual personality traits and environmental influences play a significant role in susceptibility to disordered eating, thereby underscoring the importance of personalized approaches [9,67]. Current scientific evidence recommends targeting a time in range (TIR) greater than 70% (approximately 17 h per day within the target glucose range) for most individuals with T1D, as this is associated with a lower risk of diabetes-related complications [68]. The 70% TIR benchmark is widely recognized as a clinical target, demonstrating substantial reductions in both microvascular and macrovascular complications. Although higher TIR values may provide incremental improvements in reducing the risk of long-term complications, current scientific evidence does not identify a clear point at which further complication risk reduction plateaus [69]. Consequently, effective long-term management of T1D should also focus on reducing stress related to mealtime challenges and glucose variability by fostering consistent, balanced nutrition, and avoiding excessive preoccupation with food intake. Striving for near-perfect glucose control in children and adolescents who already maintain TIR levels well above 70% may elevate the risk of DEBs as a means of coping with the perfectionism that intensive glucose management can create. It is important to communicate this risk to parents who might unintentionally encourage such behaviors by praising optimal glycemic results without recognizing the emerging underlying issues. This approach can help smooth out problems related to dietary stress and maintain healthy eating habits as part of routine diabetes care. Both ISPAD and ADA emphasize the elevated risk of DEB and clinical EDs in youth with T1D, underscoring the importance of monitoring and supportive interventions by strongly recommending comprehensive nutrition education at diagnosis and at least annually thereafter by an experienced registered dietitian to evaluate eating patterns in relation to weight status, age-appropriate growth, and cardiovascular risk factors [7]. In this context, the technological advancements in insulin administration using hybrid closed loop systems can compensate for less precise measurements (i.e through auto-corrections) and newer algorithms support simplified meal introduction (i.e small, medium. large meal) [70,71,72]. Although DEB prevalence remains high (∼42%) in pump users aged 13–21, hybrid closed-loop systems are associated lower HbA1c in DEB subgroup. More studies are needed to evaluate whether hybrid closed-loop systems may reduce the risk of DEB [73]. Table 1 presents the findings of this study compared to previously published ones.

Equally essential as monitoring for indicators of emotional or disordered eating is the implementation of early intervention, given that adolescence represents a critical period during which EDs are more likely to emerge and, in some cases, progress in severity compared to earlier childhood. Within this context, monitoring tools play a pivotal role. In 2010, Markowitz et al. developed the Diabetes Eating Problem Survey–Revised (DEPS-R), a diabetes-specific screening tool designed to identify DEBs in individuals with diabetes. This 16-item questionnaire has been shown to effectively detect individuals at elevated risk for DEB, who may subsequently benefit from psychiatric evaluation [37,74,75]. The reliability and validity of the DEPS-R were subsequently confirmed by Wisting et al., establishing its psychometric specificity [37]. A score of 20 or above indicates positive screening requiring further evaluation [37]. More recently, Calcaterra et al. suggested reorganizing the DEPS-R into four domains—restriction and body dissatisfaction, disinhibition, compensatory behaviors, and diabetes management—arguing that this factored structure provides a more accurate reflection of DEB symptomatology in the context of diabetes [76]. Another two screening tools developed over the years are the mSCOFF and the EAT26 questionnaires. The mSCOFF (modified Sick Control One Fat Food) is very brief (only 5 questions) and thus easy to integrate in everyday practice [77] while the EAT26 (Eating Attitudes Test) contains 26 items scored on a Likert scale, covering dieting, bulimia, and oral control. It is more comprehensive and better for in-depth assessment but less convenient for everyday practice [78].

Despite the fact that there are no evidence-based treatment protocols showing improvement in both DEB and glycemic outcome [79], screening done by healthcare professionals should begin in the preteen years and continue through early adulthood using validated tools like DEPS-R or mSCOFF, with quarterly reassessments by diabetologists. In low-income and low-education groups where socioeconomic barriers exacerbate poor adherence, and limited access to care exists, DEB managing becomes even more challenging and screening is extremely important for early detection. Healthcare professionals should maintain non-judgmental, supportive approaches when discussing eating concerns, as shame and fear of judgment often prevent disclosure. Red flags warranting assessment include unexplained poor glycemic control with elevated HbA1c, recurrent DKA episodes, frequent hypoglycemia after exercise, DEPS-R score ≥ 20, persistent morning hyperglycemia, significant unexplained glycemic variability, missed medical appointments, preoccupation with weight and appearance, and family reports of concerning behaviors should lead to engagement of mental health specialists [80]. This multidisciplinary collaboration between diabetes teams and mental health specialists is essential for effective identification and management and should initially involve parental education on family dynamics, followed by combined parent-youth psychoeducation or family-based therapy because apart from DEB-specific monitoring, the assessment of general psychological well-being is equally critical, as it exerts a significant influence on eating behavior [81]. Evidence indicates that difficulties in emotion regulation are positively associated with DEB among pediatric patients with T1D, with nearly half of those screening positive for DEB also exhibiting marked impairments in emotion regulation [66]. Such multidisciplinary protocols targeting optimized clinical outcomes have been described in the literature [82].

In conclusion, managing T1D in pediatric patients requires a holistic and developmentally tailored approach that addresses the distinct challenges occurring at each age. Establishing normal eating patterns, along with appropriate insulin dosing decisions, lie at the core of successful glycemic control in T1D. From infancy through adolescence, children with T1D and their caregivers must cope with a range of physical, psychological, social, and environmental issues. It is important to recognize the progressive nature of emotional disturbances into DEBs and probably into EDs and develop a strategy of early detection and intervention by including accessible technological tools, providing education and ongoing support for caregivers, identifying and addressing selective eating habits and perform systematic screening so as to ensure a smooth transition across developmental stages. In this way, healthcare providers, with the support from families, can empower young patients to manage T1D successfully, enhancing their long-term health outcomes and overall quality of life.

## Figures and Tables

**Table 1:** Comparison of this study with other previously published study on EDs and DEBs in T1D.

Study	Design	Population	Key Findings	Prevalence Rates
Current Manuscript (Scoping Review)	Scoping review including observational studies and reviews	Children (0–12 years) and adolescents (13–18 years) with T1D	Comprehensive age-specific analysis of eating behavior deviations (DEBs) and eating disorders (EDs) across developmental stages; identifies emotional eating as precursor to DEBs; insulin omission rates 19–42.4%; diabetes-specific management challenges	DEBs: up to 38% females, 16% males; pooled ED prevalence 24% (30% with >58% female participants); 32.4% with clinical ED, 8.5% with subthreshold symptoms
Colton et al. (2015) [9]	Longitudinal cohort study; follow-up from mean age 11.8 ± 1.5 years to 23.7 ± 2.1 years	126 girls with T1D over 12-year period	Long-term trajectory of ED development in females; demonstrates persistent and prevalent nature of EDs; high rates maintained into young adulthood	32.4% met diagnostic criteria for ED; 8.5% exhibited subthreshold ED symptoms
Yahia et al. (2025) [59]	Cross-sectional study on adolescent EDs in T1D	350 adolescents with T1D (12–18 years-old)	DEPS-R score significantly correlated to diabetes duration. Diabetes duration and HbA1c significant predictors of EDs	Prevalence of EDs = 22.6%. Higher prevalence of EDs in higher socioeconomic status.
Broadley et al. (2020) [65]	Narrative review exploring disordered eating in people with diabetes over the past 25 years	People with diabetes (longitudinal perspective across 25 years)	Comprehensive review of evolution of understanding regarding psychological mechanisms underlying DEBs in diabetes; identifies research gaps and methodological considerations	Girls and young women with type 1 diabetes are particularly at risk for EDs

## Data Availability

No new data were created or analyzed in this study.

## Abbreviations

The following abbreviations are used in this manuscript:

T1D	Type 1 Diabetes
T2D	Type 2 Diabetes
DEB	Disordered Eating Behavior
ED	Eating Disorder

## References

[1] Miura J., Uchigata Y. (2023). Update Information on Type 1 Diabetes in Children/Adolescents and Adults. J. Diabetes Investig..

[2] Niechciał E., Michalak M., Skowrońska B., Fichna P. (2024). Increasing Trend of Childhood Type 1 Diabetes Incidence: 20-Year Observation from Greater Poland Province, Poland. Acta Diabetol..

[3] Deeb A., Akle M., Ozairi A.A., Cameron F. (2018). Common Issues Seen in Paediatric Diabetes Clinics, Psychological Formulations, and Related Approaches to Management. J. Diabetes Res..

[4] Koller D., Khan N., Barrett S. (2015). Pediatric Perspectives on Diabetes Self-Care. Qual. Health Res..

[5] Zhou Y., Zheng M., Deng H., Zheng X., Luo S., Yang D., Mai X., Xu W., Yan J., Weng J. (2023). Relative Contributions of Fasting and Postprandial Glucose Increments, Glycemic Variability, and Non-glycemic Factors to HbA1c in Individuals with Type 1 Diabetes. J. Diabetes.

[6] Chiang J.L., Maahs D.M., Garvey K.C., Hood K.K., Laffel L.M., Weinzimer S.A., Wolfsdorf J.I., Schatz D. (2018). Type 1 Diabetes in Children and Adolescents: A Position Statement by the American Diabetes Association. Diabetes Care.

[7] Committee A.D.A.P.P., ElSayed N.A., McCoy R.G., Aleppo G., Balapattabi K., Beverly E.A., Early K.B., Bruemmer D., Echouffo-Tcheugui J.B., Ekhlaspour L. (2024). 14. Children and Adolescents: Standards of Care in Diabetes—2025. Diabetes Care.

[8] Maffeis C., Schutz Y., Fornari E., Marigliano M., Tomasselli F., Tommasi M., Chini V., Morandi A. (2017). Bias in Food Intake Reporting in Children and Adolescents with Type 1 Diabetes: The Role of Body Size, Age and Gender. Pediatr. Diabetes.

[9] Colton P.A., Olmsted M.P., Daneman D., Farquhar J.C., Wong H., Muskat S., Rodin G.M. (2015). Eating Disorders in Girls and Women with Type 1 Diabetes: A Longitudinal Study of Prevalence, Onset, Remission, and Recurrence. Diabetes Care.

[10] Seckold R., Howley P., King B.R., Bell K., Smith A., Smart C.E. (2019). Dietary Intake and Eating Patterns of Young Children with Type 1 Diabetes Achieving Glycemic Targets. BMJ Open Diabetes Res. Care.

[11] Pancheva R., Zhelyazkova D., Ahmed F., Gillon-Keren M., Usheva N., Bocheva Y., Boyadzhieva M., Valchev G., Yotov Y., Iotova V. (2021). Dietary Intake and Adherence to the Recommendations for Healthy Eating in Patients with Type 1 Diabetes: A Narrative Review. Front. Nutr..

[12] Colton P., Olmsted M., Daneman D., Rydall A., Rodin G. (2004). Disturbed Eating Behavior and Eating Disorders in Preteen and Early Teenage Girls with Type 1 Diabetes. Diabetes Care.

[13] Marks K.P., Aalders J., Liu S., Broadley M., Thastum M., Jensen M.B., Ibfelt E.H., Birkebaek N.H., Pouwer F. (2024). Associations between Disordered Eating Behaviors and HbA1c in Young People with Type 1 Diabetes: A Systematic Review and Meta-Analysis. Curr. Diabetes Rev..

[14] Ackard D.M., Vik N., Neumark-Sztainer D., Schmitz K.H., Hannan P., Jacobs D.R. (2008). Disordered Eating and Body Dissatisfaction in Adolescents with Type 1 Diabetes and a Population-based Comparison Sample: Comparative Prevalence and Clinical Implications. Pediatr. Diabetes.

[15] Wisting L., Frøisland D.H., Skrivarhaug T., Dahl-Jørgensen K., Rø Ø. (2013). Disturbed Eating Behavior and Omission of Insulin in Adolescents Receiving Intensified Insulin Treatment. Diabetes Care.

[16] Ripoli C., Ricciardi M.R., Zuncheddu E., Angelo M.R., Pinna A.P., Ripoli D. (2022). Emotional Eating and Disordered Eating Behaviors in Children and Adolescents with Type 1 Diabetes. Sci. Rep..

[17] Allen K.L., Byrne S.M., Puma M.L., McLean N., Davis E.A. (2008). The Onset and Course of Binge Eating in 8- to 13-Year-Old Healthy Weight, Overweight and Obese Children. Eat. Behav..

[18] Wheeler B.J., Lawrence J., Chae M., Paterson H., Gray A.R., Healey D., Reith D.M., Taylor B.J. (2016). Intuitive Eating Is Associated with Glycaemic Control in Adolescents with Type I Diabetes Mellitus. Appetite.

[19] Pursey K.M., Hay P., Bussey K., Trompeter N., Lonergan A., Pike K.M., Mond J., Mitchison D. (2020). Diabetes and Disordered Eating Behaviours in a Community-Based Sample of Australian Adolescents. J. Eat. Disord..

[20] Luyckx K., Verschueren M., Palmeroni N., Goethals E.R., Weets I., Claes L. (2019). Disturbed Eating Behaviors in Adolescents and Emerging Adults with Type 1 Diabetes: A One-Year Prospective Study. Diabetes Care.

[21] Young V., Eiser C., Johnson B., Brierley S., Epton T., Elliott J., Heller S. (2013). Eating Problems in Adolescents with Type 1 Diabetes: A Systematic Review with Meta-analysis. Diabet. Med..

[22] Neumark-Sztainer D., Patterson J., Mellin A., Ackard D.M., Utter J., Story M., Sockalosky J. (2002). Weight Control Practices and Disordered Eating Behaviors Among Adolescent Females and Males with Type 1 Diabetes. Diabetes Care.

[23] Troncone A., Affuso G., Cascella C., Chianese A., Pizzini B., Zanfardino A., Iafusco D., Diabetes Study Group of Italian Society of Paediatric Endocrinology and Diabetology (2022). Prevalence of Disordered Eating Behaviors in Adolescents with Type 1 Diabetes: Results of Multicenter Italian Nationwide Study. Int. J. Eat. Disord..

[24] Caccavale L.J., Nansel T.R., Quick V., Lipsky L.M., Laffel L.M.B., Mehta S.N. (2015). Associations of Disordered Eating Behavior with the Family Diabetes Environment in Adolescents with Type 1 Diabetes. J. Dev. Behav. Pediatr..

[25] Propper-Lewinsohn T., Gillon-Keren M., Shalitin S., Elran-Barak R., Yackobovitch-Gavan M., Fayman G., David M., Liberman A., Phillip M., Oron T. (2023). Disordered Eating Behaviours in Adolescents with Type 1 Diabetes Can Be Influenced by Their Weight at Diagnosis and Rapid Weight Gain Subsequently. Diabet. Med..

[26] Rosen D.S., Committee on Adolescence (2010). Identification and Management of Eating Disorders in Children and Adolescents. Pediatrics.

[27] Hanlan M.E., Griffith J., Patel N., Jaser S.S. (2013). Eating Disorders and Disordered Eating in Type 1 Diabetes: Prevalence, Screening, and Treatment Options. Curr. Diabetes Rep..

[28] Hillard J.R., Hillard P.J. (1984). Bulimia, Anorexia Nervosa, and Diabetes. Deadly Combinations. Psychiatr. Clin. N. Am..

[29] Keski-Rahkonen A., Ruusunen A. (2023). Avoidant-Restrictive Food Intake Disorder and Autism: Epidemiology, Etiology, Complications, Treatment, and Outcome. Curr. Opin. Psychiatry.

[30] Pinna F., Suprani F., Deiana V., Lai L., Manchia M., Paribello P., Somaini G., Diana E., Nicotra E.F., Farci F. (2022). Depression in Diabetic Patients: What Is the Link with Eating Disorders? Results of a Study in a Representative Sample of Patients with Type 1 Diabetes. Front. Psychiatry.

[31] Niemelä P.E., Leppänen H.A., Voutilainen A., Möykkynen E.M., Virtanen K.A., Ruusunen A.A., Rintamäki R.M. (2024). Prevalence of Eating Disorder Symptoms in People with Insulin-Dependent-Diabetes: A Systematic Review and Meta-Analysis. Eat. Behav..

[32] Butwicka A., Frisén L., Almqvist C., Zethelius B., Lichtenstein P. (2015). Risks of Psychiatric Disorders and Suicide Attempts in Children and Adolescents with Type 1 Diabetes: A Population-Based Cohort Study. Diabetes Care.

[33] Takii M., Uchigata Y., Tokunaga S., Amemiya N., Kinukawa N., Nozaki T., Iwamoto Y., Kubo C. (2008). The Duration of Severe Insulin Omission Is the Factor Most Closely Associated with the Microvascular Complications of Type 1 Diabetic Females with Clinical Eating Disorders. Int. J. Eat. Disord..

[34] Troncone A., Affuso G., Cascella C., Chianese A., Zanfardino A., Iafusco D., Diabetes Study Group of Italian Society of Paediatric Endocrinology and Diabetology (2023). Prevalence and Multidimensional Model of Disordered Eating in Youths with Type 1 Diabetes: Results from a Nationwide Population-Based Study. J. Pediatr. Psychol..

[35] Hall R., Keeble L., Sünram-Lea S.-I., To M. (2021). A Review of Risk Factors Associated with Insulin Omission for Weight Loss in Type 1 Diabetes. Clin. Child Psychol. Psychiatry.

[36] Schober E., Wagner G., Berger G., Gerber D., Mengl M., Sonnenstatter S., Barrientos I., Rami B., Karwautz A., Fritsch M. (2011). Prevalence of Intentional Under- and Overdosing of Insulin in Children and Adolescents with Type 1 Diabetes. Pediatr. Diabetes.

[37] Wisting L., Frøisland D.H., Skrivarhaug T., Dahl-Jørgensen K., Rø Ø. (2013). Psychometric Properties, Norms, and Factor Structure of the Diabetes Eating Problem Survey–Revised in a Large Sample of Children and Adolescents with Type 1 Diabetes. Diabetes Care.

[38] Ziegler A.-G., Kick K., Bonifacio E., Haupt F., Hippich M., Dunstheimer D., Lang M., Laub O., Warncke K., Lange K. (2020). Yield of a Public Health Screening of Children for Islet Autoantibodies in Bavaria, Germany. JAMA.

[39] Johnson S.B., Lynch K.F., Roth R., Schatz D., Group T.S. (2017). My Child Is Islet Autoantibody Positive: Impact on Parental Anxiety. Diabetes Care.

[40] Phillip M., Achenbach P., Addala A., Albanese-O’Neill A., Battelino T., Bell K.J., Besser R.E.J., Bonifacio E., Colhoun H.M., Couper J.J. (2024). Consensus Guidance for Monitoring Individuals with Islet Autoantibody–Positive Pre-Stage 3 Type 1 Diabetes. Diabetes Care.

[41] Chiang J.L., Kirkman M.S., Laffel L.M.B., Peters A.L., on behalf of the Type 1 Diabetes Sourcebook Authors (2014). Type 1 Diabetes Through the Life Span: A Position Statement of the American Diabetes Association. Diabetes Care.

[42] Lawton J., Waugh N., Barnard K.D., Noyes K., Harden J., Stephen J., McDowell J., Rankin D. (2015). Challenges of Optimizing Glycaemic Control in Children with Type 1 Diabetes: A Qualitative Study of Parents’ Experiences and Views. Diabet. Med..

[43] Lindström C., Åman J., Norberg A.L., Forssberg M., Anderzén-Carlsson A. (2017). “Mission Impossible”; the Mothering of a Child with Type 1 Diabetes—From the Perspective of Mothers Experiencing Burnout. J. Pediatr. Nurs..

[44] Oser T.K., Oser S.M., McGinley E.L., Stuckey H.L. (2017). A Novel Approach to Identifying Barriers and Facilitators in Raising a Child with Type 1 Diabetes: Qualitative Analysis of Caregiver Blogs. JMIR Diabetes.

[45] Hilliard M.E., Monaghan M., Cogen F.R., Streisand R. (2011). Parent Stress and Child Behaviour among Young Children with Type 1 Diabetes. Child Care Health Dev..

[46] Pierce J.S., Aroian K., Caldwell C., Ross J.L., Lee J.M., Schifano E., Novotny R., Tamayo A., Wysocki T. (2017). The Ups and Downs of Parenting Young Children with Type 1 Diabetes: A Crowdsourcing Study. J. Pediatr. Psychol..

[47] Sundberg F., Forsander G., Fasth A., Ekelund U. (2012). Children Younger than 7 Years with Type 1 Diabetes Are Less Physically Active than Healthy Controls. Acta Paediatr..

[48] Giblin S., Scully P., Dalton N., Connolly M., McCaffrey A., Sheikhi A., Neylon O., O’Gorman C. (2022). Parent and Child Perceptions of Physical Activity with Type 1 Diabetes. BMJ Open Diabetes Res. Care.

[49] Rankin D., Harden J., Barnard K., Bath L., Noyes K., Stephen J., Lawton J. (2018). Barriers and Facilitators to Taking on Diabetes Self-Management Tasks in Pre-Adolescent Children with Type 1 Diabetes: A Qualitative Study. BMC Endocr. Disord..

[50] Lowes L., Eddy D., Channon S., McNamara R., Robling M., Gregory J.W., on behalf of the Depicted Study Team (2015). The Experience of Living with Type 1 Diabetes and Attending Clinic from the Perception of Children, Adolescents and Carers: Analysis of Qualitative Data from the DEPICTED Study. J. Pediatr. Nurs..

[51] Sparapani V.d.C., Liberatore R.D.R., Damião E.B.C., Dantas I.R.d.O., de Camargo R.A.A., Nascimento L.C. (2017). Children with Type 1 Diabetes Mellitus: Self-Management Experiences in School. J. Sch. Health.

[52] Siminerio L.M., Albanese-O’Neill A., Chiang J.L., Hathaway K., Jackson C.C., Weissberg-Benchell J., Wright J.L., Yatvin A.L., Deeb L.C., Association A.D. (2014). Care of Young Children with Diabetes in the Child Care Setting: A Position Statement of the American Diabetes Association. Diabetes Care.

[53] Peeters M., Oldehinkel T., Vollebergh W. (2017). Behavioral Control and Reward Sensitivity in Adolescents’ Risk Taking Behavior: A Longitudinal TRAILS Study. Front. Psychol..

[54] Barnard K.D., Dyson P., Sinclair J.M.A., Lawton J., Anthony D., Cranston M., Holt R.I.G. (2014). Alcohol Health Literacy in Young Adults with Type 1 Diabetes and Its Impact on Diabetes Management. Diabet. Med..

[55] Mackey E.R., O’Brecht L., Holmes C.S., Jacobs M., Streisand R. (2018). Teens with Type 1 Diabetes: How Does Their Nutrition Measure Up?. J. Diabetes Res..

[56] Patton S.R. (2011). Adherence to Diet in Youth with Type 1 Diabetes. J. Am. Diet. Assoc..

[57] Gagnon C., Aimé A., Bélanger C. (2017). Predictors of Comorbid Eating Disorders and Diabetes in People with Type 1 and Type 2 Diabetes. Can. J. Diabetes.

[58] Tan J., Tan L., Davis C., Chew C. (2022). Eating Disorders in Children and Adolescents. Singap. Méd. J..

[59] Yahia S., Salem N.A., Tobar S., Abdelmoneim Z., Mahmoud A.M., Laimon W. (2025). Shedding Light on Eating Disorders in Adolescents with Type 1 Diabetes: Insights and Implications. Eur. J. Pediatr..

[60] Pinhas-Hamiel O., Hamiel U., Levy-Shraga Y. (2015). Eating Disorders in Adolescents with Type 1 Diabetes: Challenges in Diagnosis and Treatment. World J. Diabetes.

[61] Nielsen S., Emborg C., Mølbak A.-G. (2002). Mortality in Concurrent Type 1 Diabetes and Anorexia Nervosa. Diabetes Care.

[62] Gibbings N.K., Kurdyak P.A., Colton P.A., Shah B.R. (2021). Diabetic Ketoacidosis and Mortality in People With Type 1 Diabetes and Eating Disorders. Diabetes Care.

[63] Güleryüz C., Eker E., Küçükali G.K., Şakar M., Genç F.N., Şahin N.M., Elmaoğulları S., Çetinkaya S., Erdeve Ş.S. (2023). Unfavorable Effects of Low-Carbonhydrate Diet in a Pediatric Patient with Type 1 Diabetes Mellitus. J. Clin. Res. Pediatr. Endocrinol..

[64] Birkebaek N., Kahlert J., Bjarnason R., Drivvoll A., Johansen A., Konradsdottir E., Pundziute-Lyckå A., Samuelsson U., Skrivarhaug T., Svensson J. (2018). Body Mass Index Standard Deviation Score and Obesity in Children with Type 1 Diabetes in the Nordic Countries. HbA1c and Other Predictors of Increasing BMISDS. Pediatr. Diabetes.

[65] Broadley M.M., Zaremba N., Andrew B., Ismail K., Treasure J., White M.J., Stadler M. (2020). 25 Years of Psychological Research Investigating Disordered Eating in People with Diabetes: What Have We Learnt?. Diabet. Med..

[66] Yang X., Jiang H., Lin M., Yu S., Wu J. (2024). The Impact of Emotion Regulation Strategies on Disordered Eating Behavior in Children and Adolescents with Type 1 Diabetes: A Cross-Sectional Study. Front. Pediatr..

[67] Çetiner E.B., Donbaloğlu Z., Yüksel A.İ., Singin B., Behram B.A., Bedel A., Parlak M., Tuhan H. (2024). Disordered Eating Behaviors and Associated Factors in Children and Adolescents with Type 1 Diabetes. Arch. Pédiatrie.

[68] Bellido V., Pinés-Corrales P.J., Villar-Taibo R., Ampudia-Blasco F.J. (2021). Time-in-Range for Monitoring Glucose Control: Is It Time for a Change?. Diabetes Res. Clin. Pract..

[69] Bergenstal R.M., Hachmann-Nielsen E., Kvist K., Peters A.L., Tarp J.M., Buse J.B. (2023). Increased Derived Time in Range Is Associated with Reduced Risk of Major Adverse Cardiovascular Events, Severe Hypoglycemia, and Microvascular Events in Type 2 Diabetes: A Post Hoc Analysis of DEVOTE. Diabetes Technol. Ther..

[70] Rapini N., Martino M., Arnaldi C., Deodati A., Anagnostopoulou L., Amodeo M.E., Ciampalini P., Pampanini V., Lorubbio A., Tosini D. (2024). Efficacy and Safety of Advanced Hybrid Closed Loop Systems in Children with Type 1 Diabetes Younger than 6 Years. Front. Endocrinol..

[71] Ware J., Boughton C.K., Allen J.M., Wilinska M.E., Tauschmann M., Denvir L., Thankamony A., Campbell F.M., Wadwa R.P., Buckingham B.A. (2022). Cambridge Hybrid Closed-Loop Algorithm in Children and Adolescents with Type 1 Diabetes: A Multicentre 6-Month Randomised Controlled Trial. Lancet Digit. Health.

[72] Laesser C.I., Piazza C., Schorno N., Nick F., Kastrati L., Zueger T., Barnard-Kelly K., Wilinska M.E., Nakas C.T., Hovorka R. (2025). Simplified Meal Announcement Study (SMASH) Using Hybrid Closed-Loop Insulin Delivery in Youth and Young Adults with Type 1 Diabetes: A Randomised Controlled Two-Centre Crossover Trial. Diabetologia.

[73] Propper-Lewinsohn T., Elran-Barak R., Gillon-Keren M., Yackobovitch-Gavan M., Liberman A., Phillip M., Shalitin S. (2024). Disordered Eating Behaviors Among Adolescents and Young Adults with Type 1 Diabetes Treated with Insulin Pumps and Hybrid Closed-Loop Systems. Diabetes Technol. Ther..

[74] Markowitz J.T., Butler D.A., Volkening L.K., Antisdel J.E., Anderson B.J., Laffel L.M.B. (2010). Brief Screening Tool for Disordered Eating in Diabetes. Diabetes Care.

[75] Ryman B., MacIsaac J., Robinson T., Miller M.R., Gallego P.H. (2019). Assessing the Clinical Utility of the Diabetes Eating Problem Survey-revised (DEPS-R) in Adolescents with Type 1 Diabetes. Endocrinol. Diabetes Metab..

[76] Calcaterra V., Mazzoni C., Ballardini D., Tomba E., Zuccotti G.V., Mameli C., Giuseppe R.D., Cena H. (2020). Disturbed Eating Behaviors in Youth with Type 1 Diabetes: An Exploratory Study about Challenges in Diagnosis. Diagnostics.

[77] Zuijdwijk C.S., Pardy S.A., Dowden J.J., Dominic A.M., Bridger T., Newhook L.A. (2014). The mSCOFF for Screening Disordered Eating in Pediatric Type 1 Diabetes. Diabetes Care.

[78] Garner D.M., Olmsted M.P., Bohr Y., Garfinkel P.E. (1982). The Eating Attitudes Test: Psychometric Features and Clinical Correlates. Psychol. Med..

[79] Clery P., Stahl D., Ismail K., Treasure J., Kan C. (2017). Systematic Review and Meta-analysis of the Efficacy of Interventions for People with Type 1 Diabetes Mellitus and Disordered Eating. Diabet. Med..

[80] Criego A., Crow S., Goebel-Fabbri A.E., Kendall D., Parkin C. (2009). Eating Disorders and Diabetes: Screening and Detection. Diabetes Spectr..

[81] Goebel-Fabbri A.E., Uplinger N., Gerken S., Mangham D., Criego A., Parkin C. (2009). Outpatient Management of Eating Disorders in Type 1 Diabetes. Diabetes Spectr..

[82] Versloot J., Ali A., Minotti S.C., Ma J., Sandercock J., Marcinow M., Lok D., Sur D., de Wit M., Mansfield E. (2021). All Together: Integrated Care for Youth with Type 1 Diabetes. Pediatr. Diabetes.

